# The effect of parenting behaviours on adolescents’ rumination: a systematic review of longitudinal studies

**DOI:** 10.1007/s00787-023-02309-2

**Published:** 2023-10-09

**Authors:** Tiago Castro, Tiago Miguel Pinto, Ana Morais, Raquel Costa, Inês Jongenelen, Diogo Lamela

**Affiliations:** 1 HEI-Lab, Lusófona University, Porto, Portugal; 2 EPIUnit, Instituto de Saúde Pública, Universidade do Porto, Porto, Portugal; 3 Laboratório para a Investigação Integrativa e Translacional em Saúde Populacional (ITR), Porto, Portugal

**Keywords:** Rumination, Parenting, Adolescents, Children, Child maltreatment, Emotion regulation

## Abstract

Rumination is an emotional regulation mechanism strongly associated with the development and maintenance of internalising psychopathology in adolescence and adulthood. Parenting behaviours (PBs) play a pivotal role in the development of rumination in children and adolescents. Nonetheless, the specific PBs that can either protect against or increase the risk of rumination development remain poorly understood. This systematic review aimed to explore the (1) temporal associations between PBs and adolescents’ rumination and (2) potential moderators influencing these associations. We conducted a comprehensive search across Web of Science, Scopus, PubMed, Academic Search Complete and Eric databases, adhering to PRISMA reporting guidelines. Out of 1,868 abstracts screened, 182 articles underwent full-text examination, with nine meeting the inclusion criteria for the systematic review. Overall, the studies indicated that PBs characterised by criticism, rejection and control were positively associated with the development of rumination in adolescents, whilst PBs marked by authoritative practises exhibited a negative association with rumination. Gender, temperament, environmental sensitivity and pubertal timing emerged as significant moderators in the effects of PBs on rumination. However, conclusions were limited due to the studies’ methodological heterogeneity. Future studies on PBs and rumination should address various dimensions of PBs and different moderators to identify factors that can modify the development of rumination across adolescence. Findings may inform family-based prevention programmes to promote emotion regulation in adolescents as a protective factor against internalising psychopathology across adulthood.

## Introduction

The Response Styles Theory [1, 2] defines rumination as a response to negative affect through a systematic reflection upon the symptoms, causes and consequences of the emotional state. Individuals who ruminate are less invested in actively solving their problems, leading them to behavioural inaction [3]. Several attempts have been made to explain why humans engage in such thinking styles. Behaviourists claim that ruminative thinking works as a tool for individuals to stay away from aversive situations [4] as it seems to usually appear in those who are exposed to punitive environments [5]. This emotion regulation mechanism helps to create a rationale that repeatedly communicates the uselessness of trying to actively solve the problem, inducing subsequent inactivity [3]. On the other hand, evolutionary theories claim that withdrawal symptoms, such as social disengagement, work towards preserving resources that would otherwise be threatened if one was actively involved in the situation. The engagement in rumination collects information that serves as evidence for the hopelessness in problem-solving [3], leading to withdrawal and, thereof, the preservation of resources.

This form of rumination is usually known as depressive or negative rumination due to its focus on negative affect [6]. Yet, there are other forms of this self-regulatory mechanism, such as anger and positive rumination [7, 8]. Anger rumination is also characterised by repetitive thoughts, but in this case, these are focussed on anger-related experiences [8]. As for positive rumination, Feldman et al. [7] defined the construct as a response to positive affect with thoughts revolving around positive aspects about oneself.

Rumination has been consistently linked with internalising symptomatology and is thought of as a transdiagnostic feature for depression and anxiety [9–12]. In a meta-analysis focussed on the effect of emotion regulation mechanisms on psychopathology symptoms, rumination was found to have the most significant positive effect size, compared to avoidance, problem-solving, suppression, reappraisal and acceptance [13]. Other studies also found a positive association between rumination and anxiety [14]. These data suggest that rumination is a robust risk factor for developing internalising psychopathology.

Caregivers can serve as models to their children [15] and PBs are potential predictors of children’s failure to develop effective emotion regulation mechanisms to cope with negative emotions [3, 15, 16]. Therefore, parents that often engage in criticism can promote self-criticism in the child. This can lead to low self-efficacy and, thereafter, a marked disengagement in problem-solving. Due to hopelessness when facing a problem, the child can resort to passive cognitive strategies such as rumination [17]. The Response Styles Theory [18, 19] adds to this rationale by advocating that lower parental efforts in promoting child autonomy in engagement with the world might lead to the adoption of passive approaches, such as rumination, which can impair the active development of problem-solving skills. In agreement with these conceptual models, empirical studies found that overcontrolling and negative parenting can lead to higher levels of rumination in childhood and adolescence [20, 21].

According to the attachment theory [22], PBs marked by rejection do not fulfil the children’s needs for carrying and safety, which is associated with developing an insecure attachment characterised by negative representations of the self and others. These children are more prone to search for cues of negative affect and ruminate about them [23]. Rumination can be an attempt to preserve proximity to one’s caregivers [24], and if proven useful, can be generalised to other contexts [25]. Hostile PBs, such as rejecting parenting, were highly associated with decreased emotion regulation in children [26], and specifically, maternal withdrawal coping mechanisms predicted increased levels of rumination in adolescents [27]. In addition, maltreating practises perpetrated by caregivers usually set an environment characterised by punishment [28], preventing the child from actively solving a problem. Previous research investigated the role of maltreating practises as a potential inflictor of maladaptive emotion regulation mechanisms, namely rumination [29–31].

There is currently no systematic review in the existing literature examining the association between PBs and the development of rumination. Resembling it, Cortés-García et al. published a meta-analysis accounting for the mechanisms underlying the relationship between attachment insecurity and depressive symptoms [32]. Their findings suggested that the brooding dimension of rumination significantly mediated this relationship. Even though an insecure attachment is mostly developed through PBs such as rejecting, overcontrolling and criticising [33], it does not give a direct account of how PBs are associated with rumination.

The primary objective of this systematic review was to comprehensively investigate the temporal association between PBs and the development of rumination, aiming to enhance our understanding of this phenomenon. Whilst cross-sectional studies have been instrumental in identifying potential PBs associated with rumination amongst adolescents, the utilisation of longitudinal designs offered a distinct advantage by establishing temporal relationships between potential PB predictors and the emergence of rumination, as hypothesised by existing conceptual models. Furthermore, a noteworthy body of prior research has suggested that this association is influenced by gender and temperament-related factors [9, 20, 34]. As such, this review sought to explore whether certain children exposed to negative PBs exhibit greater vulnerability in the development of rumination compared to their counterparts.

Due to sufficient empirical evidence demonstrating the significance of the impact different PBs have on the development of rumination in adolescents [20, 34, 35], this systematic review aims to explore the (1) temporal association between PBs and children’s/adolescents’ rumination and (2) potential moderators of this association. The following questions were addressed:

1. Which PBs are associated with increased levels of adolescents’ rumination?
2. Is this association mostly significant?
3. Which variables potentially moderate this effect?

## Methods

For this review, the Preferred Reporting Items for Systematic Reviews and Meta-Analyses (PRISMA) guidelines were used [36].

## Data sources

The scientific search engines used to identify articles on the association between PBs and child/adolescent rumination, and potential moderators were Web of Science, Scopus, PubMed, Academic Search Complete and Eric. There were no restrictions on publishing dates.

## Search terms

The grouped terms used were the following: (“rumination”) AND (“parenting” OR “parental behaviour” OR “parents” OR “mother” OR “father” OR “maternal” OR “paternal” OR “parental” OR “transmission”).

## Eligibility criteria

The review included longitudinal and quantitative studies that met the following inclusion criteria:

1. Used at least one measure of negative rumination
2. Had a sample of children or adolescents till 20 years of age at the last assessment wave of the study
3. Reported data about at least one primary caregiver
4. Reported on at least one measure of PB
5. Explored the temporal effect of PBs on children’s/adolescents’ rumination

Studies were excluded if:

1. They considered emotion regulation instead of rumination, specifically
2. Not written in English, Spanish, Portuguese, or German language
3. Unpublished
4. They were book chapters, dissertations summaries, or conferences
5. Systematic reviews or meta-analyses

## Screening procedure

The titles and abstracts from the initial search were independently read by T.C. and D.L. to screen for full-text retrieval. Whenever a title seemed relevant, but no abstract was available, the full text was retrieved. The review’s final decision on eligibility for inclusion resulted from an independent screen of all full-text articles by T.C., A.M., and D.L.

## Data extraction

Data extraction was conducted according to the aims of this systematic review. This extraction resulted in the gathering of information concerning the first author’s name, the date and country of study, the characteristics of the sample (i.e. age mean and the population it represents), the measures used to assess PBs and the child/adolescent rumination and the study’s main aim and design.

## Data synthesis

The reviewed studies were qualitatively synthesised. To address the aims of this review, the relevant findings of the included studies were codified into three major sections: 1) PBs (e.g. overcontrolling parenting, parenting styles, parental affective expression and maltreatment exposure and severity); 2) rumination (i.e. adolescent rumination, child rumination and sadness rumination); and 3) moderators of the association between PBs and child/adolescent rumination (i.e. gender and temperament).

## Results

### Included articles

**Fig. 1:**
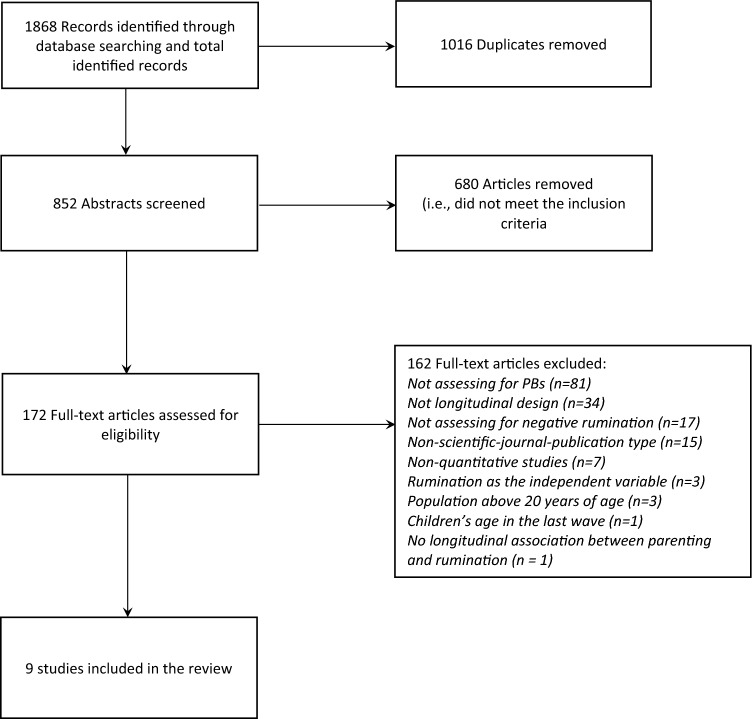
Flowchart of the article search and selection process (Source: PRISMA, Moher et al., 2009)

The database search resulted in the retrieval of 1,868 records (see Fig. 1). After the abstract screening, 182 articles were considered for full-text reading. From full-text screening, nine studies were eligible and included. Every disagreement between independent researchers was discussed to the point of resolution. The main exclusion reasons were the assessment of an independent variable rather than PBs, the non-longitudinal design of the study, the assessment of a dependent variable rather than negative rumination and non-scientific journal publication. All the included articles explore temporal associations between PBs with adolescents’ rumination. Furthermore, this analysis incorporates all of the rumination assessment instruments employed in the reviewed articles.

### Study characteristics

As shown in Table 1, children/adolescents samples’ age ranged from 1 to 13 years old at the first assessment wave. Due to the longitudinal design, some studies cover the whole age range to explore the temporal associations between PBs and adolescents’ rumination. One study did not rely on any caregiver to assess PBs [37]. Regarding the study design, there were five studies with three assessment waves [34, 35, 37–39], three studies with two assessment waves [20, 40, 41] and one with four assessment waves [42]. Six studies were conducted in English-speaking countries, such as Australia [35], Honk Kong [40] and the US [20, 34, 41, 42] (see Table 1). Finally, there was one study from China [38], one from Spain [37] and one from Finland [39]. All studies examined PBs exclusively during the initial assessment wave, except for Tammilehto et al. [41], who conducted assessments of PBs across all three waves of the study. Regarding rumination, seven studies measured it in only one assessment wave, whilst the remaining studies assessed it on two measurement moments [35, 40].

**Table 1:** Summary of studies of PBs and children rumination

First author (year)	Country	Child/adolescent sample and age at baseline	Parenting dimension	Parenting behaviour assessment (procedure; wave)	Child/adolescent rumination assessment (procedure; wave)	Sample setting	Longitudinal design (mean time between waves)	Main aim
Dunning et al. (2022)	USA	629 adolescents (*M* = 13.05 years, *SD* = 0.90)	1. Psychological autonomy vs. control 2. Family communication, affective expression and involvement	1. The subscale autonomy vs. psychological control of the CRPBI (parent report; W2) 2. FAM-III-G (parent report; W2)	CRSQ (self-report; W3)	Community	4-wave (W1–W2: 1.68 years; W2–W3: 1.16 years; W3–W4: 0.37 years)	To explore the development of rumination and its relationship with internalising psychopathology
Gaté et al. (2013)	Australia	163 adolescents (*M* = 12.46 years, *SD* = 0.43)	1. Aggressive and positive interpersonal behaviour	1. LFE (observational; W1)	RRS of the RSQ (self-report; W1 and W2)	At risk	3-wave (W1–W2: 2.5 years; W2W3: 1.6 years)	To examine whether rumination mediates the relationship between a negative family environment and adolescent depressive symptoms
Hilt et al. (2012)	USA	337 children (4½ years, *SD* = not reported)	1. Overcontrolling parenting 2. Negative-submissive family expressivity	1. CRPR (parent report; W1) 2. FEQ (parent report; W1)	RRS (self-report; W2)	Clinical	2-wave (W1–W2: between 13.5 and 15.5 years)	To examine the moderation effect of effortful cognitive control on the relationship between early family environment and the development of adolescent rumination
Li et al. (2021)	China	950 adolescents (*M* = 13.18 years, *SD* = 0.66)	1. Parental solicitation	1. 5 items of the PKS (parent report; W1)	SRI (self-report; W2)	Community	3-wave (1 year between each wave)	To examine the moderation effect of both sadness rumination and parental solicitation on the relationship between pubertal timing and adolescent depressive symptoms
Lionetti et al. (2021)	USA	292 children (*M* = 3.70 years; *SD* = 0.26)	1. Parenting styles: permissive, authoritarian and authoritative	1. PSDQ (parent report; W1)	RRS (self-report; W2)	Community	3-wave (W1–W2: 6 years; W2–W3: 3 years)	To explore the effect parenting styles, emotional sensitivity and both combined have on the development of depression in the child with child rumination mediating these relationships
Lo et al. (2021)	Hong Kong	125 adolescents (*M* = 12.21 years, *SD* = 1.39)	1. Parental demandingness	1. PCIT (observational; W1)	CRSQ (self-report; W1, W2)	Community	2-wave (W1–W2: 1 year)	To examine the association between parental demandingness and adolescent depression by looking into the mediating effect rumination might have on this relationship
Padilla Paredes et al. (2014)	Spain	1,316 adolescents (*M* = 13.42 years, *SD* = 1.3)	1. Emotional abuse by parents	1. CTS-PC Spanish version (self-report; W1)	Ruminative Responses Subscale of the CRSS (self-report; W2)	Community	3-wave (W1–W2: 6 months; W2–W3: 6 months)	To examine the involvement of cognitive mechanisms in the relationship between parental and peer abuse, and adolescent depression
Schweizer et al. (2018)	USA	425 children (*M* = 3.5 years, *SD* = 0.3)	1. Positive parenting	1. TTB (observational; W1)	Ruminative Response subscale of the CRSQ (self-report; W2)	Community	2-wave (W1–W2: 6 years)	To examine the effect of early childhood temperament and PBs on the development of children’s ruminative thinking style
Tammilehto et al. (2021)	Finland	885 children (*M* = 1 year, *SD* = not reported)	1. Parenting quality	1. Parental autonomy and intimacy subscales of the SFPT (parent report; W1, W2, W3)	Rumination subscale of the CERQ (self-report; W3)	Community	3-wave (W1–W2: 6.5 years; W2–W3: 11 years)	To examine the stage-specific effects of parenting on adolescents’ emotion regulation

Note**:** *CERQ* Cognitive Emotion Regulation Questionnaire, *CRPBI* Children’s Report of Parenting Behaviour Inventory, *CRPR* Child-Rearing Practises Report, *CRSQ* Child Responses Style Questionnaire, *CRSS* Children’s Responses Style Scale, *CTS-PC* Conflict Tactics Scales—Parent to Child, *FAM-II-G* Family Assessment Measure III—General, *FEQ* Family Expressiveness Questionnaire, *LEQ* Lifetime Experiences Questionnaire *LFE* Living in Familial Environments, *M* Mean, *PCIT* Parents–Child Interaction Task, *PKS* Parent Knowledge Scale, *PSDQ* Parenting Styles and Dimensions Questionnaire, *RRS* Ruminative Responses Scale *RSQ* Responses Style Questionnaire *SD* Standard Deviation, *SFPT* Subjective Family Picture Test, *SRI* Sadness Rumination Inventory, *TTB* Teaching Tasks Battery

### Effect of PBs on adolescents’ rumination

Table 2 provides a comprehensive overview of the assessment tools employed in the reviewed studies to measure rumination. A total of five distinct self-report measures were utilised for this purpose. The Ruminative Responses Subscale of the Children’s Response Styles Questionnaire (CRSQ-R; [43]) and the Ruminative Responses Scale (RRS; [44]) emerged as the most commonly utilised instruments. All these measures have examined their psychometric properties and suitability for assessing rumination.

**Table 2:** Description of the child/adolescent rumination assessment tools

Child/adolescent rumination assessment tool	Studies
The Ruminative Responses Subscale of the Children’s Response Styles Questionnaire (CRSQ-R; [43]) is a self-report assessment tool that measures children’s ruminative response to depressed mood. The subscale includes 13 items (e.g. “Think about how alone you feel”) with answers ranging from 0 (i.e. “almost never”) to 3 (i.e. “almost always”) in a Likert scale	Dunning et al. (2022) Lo et al. (2021) Schweizer et al. (2018)
The Ruminative Responses Scale (RRS; [44]Nolen-Hoeksema & Morow, 1991) of the Response Styles Questionnaire is a self-report assessment tool that measures self-, symptoms-, and causes and consequences-focussed responses to depressed mood. The subscale includes 22 items (e.g. “I think back to other times I have been depressed”) with answers ranging from 1 (i.e. “almost never”) to 4 (i.e. “almost always”) in a Likert scale	Gaté et al. (2013) Hilt et al. (2012) Lionetti et al. (2021)
A brief version of the Sadness Rumination Inventory (SRI; [55]) is a self-report assessment tool that measures adolescents’ sadness rumination. It includes 11 items (e.g. “When I feel sad, the more I think of it, the sadder I become”) with answers ranging from 1 (i.e. “never”) to 5 (i.e. “always”) in a Likert scale	Li et al. (2021)
The Spanish adaptation of the Ruminative Responses Subscale of the Children’s Response Styles Scale (CRSS; [56]) is a self-report assessment tool that measures brooding and reflection rumination. It includes 10 items (e.g. “I think back to other times when I felt this way”) with answers ranging from 1 (i.e. “almost never”) to 4 (i.e. “almost always”) in a Likert scale	Padilla Paredes et al. (2014)
The Rumination Subscale of the Cognitive Emotion Regulation Questionnaire (CERQ; [57]) is a self-report assessment tool that measures rumination as a cognitive strategy of emotion regulation. It includes 4 items (e.g. “I dwell upon the feelings the situation has evoked in me”)	Tammilehto et al. (2021)

As illustrated in Table 1, the studies included in this review assessed a range of PBs, encompassing psychological autonomy and control, family communication, affective expression, parenting involvement, aggressive and positive interpersonal behaviours, negative-submissive expressivity, overcontrol, positive parenting, parenting styles (i.e. permissive, authoritarian and authoritative), parental solicitation, demandingness, emotional abuse and parenting quality. Table 3 offers comprehensive definitions for each of these parenting behaviours, drawing from either the definitions utilised in the respective articles or those adopted by the measures employed in the studies to operationalise the parenting behaviour constructs under investigation. In addition, several moderators influence the relationship between PBs and rumination, including inhibitory control, negative affect, effortful control, gender, pubertal timing and environmental sensitivity.

**Table 3:** Definitions for each parenting dimension

First author (year)	Parenting dimension
Dunning et al. (2022)	Psychological autonomy vs. control: a parental practise that enables children to explore and enact their personal desires and wishes vs. a parental practise that aims to control and manipulate children’s thoughts and emotions Family communication: quality and quantity of communication and mutual understanding between family members Affective expression: the ability to communicate and express emotions amongst family members Involvement: the time and quality of family members’ interest in one another
Gaté et al. (2013)	Aggressive interpersonal behaviour: verbal and non-verbal aggressive behaviours (e.g. being angry, disapproving of one’s behaviour, threatening and arguing against) parents have towards their children in direct interaction Positive interpersonal behaviour: verbal and non-verbal positive behaviours (e.g. validating, approving and caring) parents have towards their children in direct interaction
Hilt et al. (2012)	Overcontrolling parenting: a parental practise that aims to control and manipulate children’s thoughts, emotions and behaviours Negative-submissive family expressivity: frequent expression of emotions such as sadness, guilt and embarrassment as a coping mechanism
Li et al. (2021)	Parental solicitation: a parental practise that involves the active seeking of information regarding their children
Lionetti et al. (2021)	Permissive parenting: non-demanding, child-driven, and does not apply rules to the child Authoritarian parenting: strict, controlling, restrictive and implementing non-negotiable rules Authoritative parenting: nurturing, responsive and supportive
Lo et al. (2021)	Parental demandingness: the degree to which parents set boundaries for their children's behaviour and impose consequences for the violation of these boundaries
Padilla Paredes et al. (2014)	Emotional abuse: this term describes a situation in which parents create an environment in which their children experience negative self-appraisal, such as feeling humiliated, worthless or belittled
Schweizer et al. (2018)	Positive parenting: high levels of positive affectivity expression (e.g. smiling, approving or kissing), parental support (expressions of positive consideration towards the child) and relationship quality (positive interactions between the dyad parent–child), combined with low levels of negative affective expression (e.g. anger, shouting or cursing) and parental hostility (frustration and rejection towards the child)
Tammilehto et al. (2021)	Parenting quality: the parent–child interaction is marked by autonomy and intimacy

#### Parenting control and demandingness

Five studies examined parenting control, encompassing psychological and behavioural control. Dunning et al. [42] concluded that mothers' psychological control when adolescents were 13 years old (wave 2) was not associated with adolescent rumination 1 year later (wave 3). Tammilehto et al. [39] found that higher levels of parental autonomy, as self-reported by fathers and mothers when the child was 1 year old (wave 1), and as self-reported by fathers (but not mothers) when the child was 7 to 8 years old (wave 2), were associated with lower levels of children's rumination during ages 17 and 19 (wave 3). No concurrent associations were found between parental autonomy reported by fathers and mothers and rumination at assessment wave 3. In addition, no associations between mothers' and fathers' intimacy parenting style (i.e. parenting behavioural control) and rumination were found in this study.

The remaining two studies found a positive association between parenting control/demandingness and rumination [20, 40]. Lo et al. [40] reported that higher levels of parental demandingness at an average age of 12 years (wave 1) were associated with higher levels of rumination 1 year later (wave 2). However, no concurrent association between parental demandingness and rumination was found at age 12. Hilt et al. [20] also concluded that higher levels of overcontrolling parenting at the age of 4½ years (wave 1) were associated with higher levels of rumination in the assessment wave when adolescents were aged between 13 and 15 years (wave 2).

#### Negative-submissive family expressivity and parental affective expression

Children aged 4½ (wave 1) who experienced increased negative-submissive family expressivity exhibited significantly higher levels of adolescent rumination when they reached 13 and 15 years of age (wave 2) [20]. Conversely, Dunning et al. [42] found that the mother's affective expression when the adolescents were 15 years old (wave 2) was not linked to adolescents' rumination 1 year later (wave 3).

#### Parental communication, involvement and solicitation

In the study by Dunning et al. [42], it was discovered that family communication and involvement, measured when adolescents were 13 years old (wave 2), were not linked to adolescent rumination 1 year later (wave 3). Likewise Li et al. [38] did not identify an association between parents' self-reported parental solicitation (a dimension of parental monitoring) when children were 13 years old (wave 1) and adolescent rumination 1 year later (wave 2).

#### Parental interpersonal behaviours and positive parenting

In the study conducted by Gaté et al. [35], no significant association was observed between aggressive and positive parent–child behaviours during event-planning and problem-solving interactions when children were 12 years old (wave 1) and adolescent rumination at 15 years old (wave 2). In contrast, Schweizer et al. [41] concluded that higher levels of positive parenting when children were aged 3 (wave 1) were linked to lower levels of rumination when they reached 9 years old (wave 2).

#### Parenting styles

In Lionetti’s et al. study [34], permissive parenting at age 3 (wave 1) was not found to have a temporal association with children's rumination at age 9 (wave 2). Conversely, authoritarian parenting at age 3 was linked to higher levels of rumination at age 9, whilst authoritative parenting exhibited a negative association with rumination within the same timeframe.

#### Maltreating practises

In the study conducted by Padilla Paredes and Calvete [37], a positive association was discovered between parental emotional abuse when children were 13 years old (wave 1) and both brooding and reflection rumination 6 months later (wave 2).

## Potential moderators of the effect of PBs on adolescents’ rumination

### Child’s temperament

Hilt et al. [20] determined that children's negative affect and effortful control played significant moderating roles in the relationship between overcontrolling parenting at the age of 4½ years (wave 1) and child rumination at ages 13 to 15 years (wave 2). Specifically, the association between overcontrolling parenting (wave 1) and increased rumination (wave 2) was statistically significant only amongst the group of adolescents with high levels of negative affect or effortful control at age 4½ years. Furthermore, the child's negative affect also significantly moderated the relationship between negative-submissive family expressivity at wave 1 and child rumination at ages 13 to 15 years (wave 2). In this case, the association between negative-submissive family expressivity (wave 1) and increased rumination (wave 2) was significant solely amongst the group of adolescents with low levels of negative affect at the age of 4½ years.

Schweizer et al. [41], on the other hand, observed that the relationship between positive parenting at age 3 (wave 1) and rumination at age 9 (wave 2) was moderated by inhibitory control. Specifically, the significant association between positive parenting and rumination was evident only when inhibitory control was high.

### Gender

Only one study formally examined the moderating effect of gender between PBs and rumination. Gaté et al. [35] found that the association between positive maternal behaviours assessed at the child’s age of 12 (wave 1) and decreased rumination at 15 years (wave 2) was significant only in girls.

### Environmental sensitivity

Lionetti et al. [34] observed that environmental sensitivity was a significant moderator of the relationship between permissive parenting when children were 3 years old (wave 1) and children's rumination at age 9 (wave 2). Specifically, the association between permissive parenting at age 3 and rumination at age 9 was significant only amongst children with high levels of environmental sensitivity. For both authoritarian and authoritative parenting styles, interactions with rumination were not influenced by environmental sensitivity.

### Pubertal time

Li et al. [38] identified pubertal timing as a significant moderator in the relationship between parental solicitation when children were aged 13 (wave 1) and rumination 1 year later in girls. More precisely, the influence of parental solicitation on rumination was more pronounced in girls with earlier pubertal timing compared to those with later pubertal development.

## Discussion

The focus of this systematic review was to analyse longitudinal studies that explore the effects of PBs on children/adolescents’ rumination, as well as the potential moderators of this effect. Our systematic review included empirical studies that focussed on multiple domains of PBs, using different assessment methods (i.e. self-reports, observations and interviews), samples and children’s/adolescents’ ages.

The association PBs have with rumination tends to vary depending on the assessed PB. Parenting control [20, 40], negative-submissive family expression [20], negative affectivity [41], authoritarian parenting [34] and emotional abuse [37] are associated with rumination in adolescents. These associations suggest that these PBs may constitute a risk factor for the development of this emotion regulation mechanism. In general, the results of the studies here included were dominated by positive associations. Yet, this might be due to the aims of the studies, since most of them intend to corroborate the existence of positive associations between specific PBs and rumination.

Data from eligible studies indicated that positive affectivity [41], authoritative parenting [34], low control [39] and parental solicitation (a dimension of parental monitoring) [38] are associated with lower rumination. Interestingly, parental solicitation is conceptualised as an active investment in obtaining information about the child/adolescent and their friends [45]. In western culture, this might be deemed as a form of parenting control, and therefore, a negative PB [46]. However, Li et al.’s study [38] was conducted in Chinese culture where parental solicitation is usually perceived as a supportive form of parenting [47]. This might explain its negative association with rumination, contrary to other studies conducted in western culture and assessing other forms of parenting control [20, 40]. However, the different outcomes might also be due to the nuanced differences between parenting control and parental solicitation. The first is restrictive, critical and engaged in monitoring [20, 40], making it a broader construct, whilst the latter is mostly engaged in monitoring by actively seeking information about the child/adolescent [45].

PBs such as parental involvement, family communication, mother affective expression, rejecting parenting, maternal behaviours (i.e. positive and negative on the event-planning interaction and problem-solving interaction) and permissive parenting were not associated with rumination [34, 35, 42]. Three of these variables were from the same study (i.e. parental involvement, family communication and mother affective expression) [42]. This outcome might be explained by the fact that the study modelled and analysed these three variables equally.

Amongst the parenting dimensions associated with less rumination in adolescents, one is affective (i.e. positive affectivity), one behavioural (i.e. low control) and the other is affective behavioural (i.e. authoritative parenting) [34, 39, 41]. Regarding the parenting dimensions associated with more rumination in adolescents, three are behavioural (i.e. parenting control, maltreatment severity and exposure and emotional abuse), two affective (negative-submissive family expression and negative affectivity) and another is affective behavioural (authoritarian parenting) [20, 37, 40, 41]. The behavioural dimension seems to reflect parenting practises that are both restrictive for and critical of the adolescent, and the affective dimension is mostly reflective of practises that are critical and rejecting of the adolescent.

The Ruminative Response Style Theory [1, 6, 18] suggests that highly critical, restrictive and intrusive parenting styles lead to the children’s/adolescent’s failure to learn active emotion regulation mechanisms and to the experience of hopelessness in controlling one’s environment. Because children/adolescents do not have the chance to actively solve their problems, due to restrictions on behaviour and emotional expressivity, they end up having to resort to passive cognitive emotion regulation mechanisms such as rumination. These review’s findings seem to support this theory by suggesting that PBs of overcontrol and emotional expression restriction, which are marked by criticism, intrusiveness and restriction, are associated with rumination [20, 37, 40, 41]. The Ruminative Response Style Theory [1, 18] also suggests that rejecting PBs may be involved in the development of rumination, because of the lack of positive involvement and orientation. Children/adolescents that are left on their own to deal with their problems and emotions may feel helpless when facing distress, and therefore rely on inward thinking. Findings support this theoretical claim by suggesting that rejecting PBs such as negative-submissive family expression [20] and permissive parenting [34] are associated with rumination.

In line with the Ruminative Response Style Theory [1, 18], nurturing, responsive and supportive PBs should set an environment that encourages engagement in a wide range of behaviours and emotions that lead to the development of problem-solving mechanisms. The reviewed findings seem to corroborate this assumption since low parenting control, high positive parental emotional expression and the exercise of authoritative parenting styles are associated with low rumination [34, 39, 41].

Effortful control, negative affectivity, inhibitory control, gender and environmental sensitivity were significant moderators of the relationship between PBs and rumination. The Ruminative Response Style Theory [1, 18] hypothesised that a difficult temperament and gender are associated with the development of rumination. It suggests that reactive individuals may consider states of negative affect more enthralling and, therefore, be more prone to direct their attention towards them. When comparing themselves to less-reactive individuals, who do not seem as often triggered, they may start questioning their emotional reactions. Also, children/adolescents that constantly direct their attention to their negative emotions and to the questioning of their affective states may develop rumination as a recurrent reaction to negative affect.

Similarly, children or adolescents with more effortful control can change their emotional and behavioural responses by directing their attention away from negative emotional states [48]. However, emotion regulation mechanisms partially develop through parenting encouragement [49]. In a high parenting control context, adolescents with high levels of effortful control might miss out on this encouragement and end up orienting their ability to sustain their attention towards inwards-directed emotion regulation mechanisms, such as rumination. Additionally, individuals more prone to negative affect and/or with higher environmental sensitivity seem to be more likely to focus their attention on negative emotional states and to try to make sense of them [1, 18]. People with high environmental sensitivity tend to avoid direct and instant engagement with new environments so that they can process the information in their own time [50]. This translates into behavioural inactivity, which is characteristic of the concept of rumination laid out by the Ruminative Response Style Theory [1, 18]. Regarding gender, parents might punish boys’ engagement in emotional expression based on their own gender expectations, and therefore make them engage in distracting responses when faced with a negative mood. This might promote the development of effortful control in boys and not in girls [1, 18]. Also, Pomerantz et al. [51] saw that parents exert more control practises on girls than on boys, making girls feel behaviourally restricted, and consequently resort to cognitive strategies such as rumination. The Diathesis-Stress Model of Environmental Action [52] also adds to this rationale by saying that individual differences, such as temperamental (e.g. effortful control) and genetic/identity (e.g. female gender) differences, influence how a person responds to the environment. Findings seem to support all of the above by showing that gender, effortful control, negative affect and environmental sensitivity moderate the relationship between PBs and children’s/adolescent’s rumination [20, 34, 41].

## Strengths and limitations of the reviewed studies

The studies included in this review have strengths and limitations. The studies had several methodological dissimilarities, which constituted a limitation in interpreting their results. The assessment methods used for the parenting construct were distinct (i.e. observational, child-report, parent-report, interview), compromising comparisons across studies. The studies relied mostly on self-reports [20, 34, 37–39, 42], which largely depend on the accuracy of the participants’ recall memory. However, some studies had multiple methods of assessment [35, 40, 41] supplied by different informants (e.g. parents, children, adolescents and partners), which avoids the risk of reporting bias susceptible to studies that exclusively use questionnaires. There was also a heterogeneity across studies in the assessed parenting construct, accounting for various forms of parenting such as parental communication, parental affective expression, parental involvement [42], maternal behaviours [35], overcontrolling parenting, negative-submissive family expressivity [20], parental solicitation [38], parental demandingness [40] and emotional abuse [37]. Parenting control was explored in four different studies [20, 39, 40], whereas the remaining were only assessed by one study. This makes parenting control the only construct susceptible to interstudy comparison.

The studies also differed regarding the number of follow-ups after baseline assessments, the time periods between the follow-ups and the children’s/adolescents’ age. Hampel et al. [53] found an increase in rumination from ages 8 to 13 years, which suggests that the development of rumination might depend on a specific stage of development. However, some studies assessed it only throughout one developmental stage [34, 41] and others both in childhood and adolescence [39], precluding the possibility of interpreting age-specific results. Whilst all these differences might induce variability in the results, the findings were relatively consistent across the studies, such that the parenting construct interacted significantly with rumination. Regarding strengths, two out of three moderators (i.e. effortful control and gender) were assessed in different studies [20, 35, 41], making it possible to perform interstudy comparisons.

## Future directions

In the future, more studies should account for repeated intra-individual measurements to identify differential age effects. It is also relevant that future research uses multiple methods of assessment to avoid reported bias characteristic of exclusive questionnaire use. Most research is focussed on exploring the effect of PBs and rumination on the development of depressive symptomatology [18, 32, 54]. Given the extensive literature corroborating this effect, it is important to explore the mechanisms underpinning the development of rumination. We also recommend that future research accounts for mediators and other possible moderators of the effect of PBs on rumination. This is crucial to identify who is more prone to develop the emotion regulation mechanism and which factor provoked by PBs is exacerbating its levels. Moreover, future longitudinal studies would benefit from the inclusion of cross-lag analyses. These analytical methods offer the capacity to explore causal links, shedding light on whether specific parenting behaviours precede heightened child rumination or if child rumination influences subsequent parenting practises. Additionally, cross-lag analyses enable the examination of potential bidirectional influences, revealing the reciprocal impact of these variables over time.

Since there is a wide variety of PBs and various ways of conceptualising them, it is important that there are multiple data on all these variances. This will allow research to converge into the PBs that seem to have a strong and replicable effect on rumination. Finally, future research should be focussed on both parents and not just on mothers or female caregivers.

## Clinical implications

This systematic review can contribute to a better understanding of the role of PBs in the development of rumination. Since this emotional regulatory mechanism is strongly linked to internalising psychopathology [9, 11, 12], an understanding of how it develops can be crucial to prevent and intervene in internalising psychopathology in children and adolescents. The reviewed studies support that specific PBs are associated with the development of children’s/adolescents’ rumination. It is, therefore, relevant to implement detection (e.g. structured clinical interviews and assessment instruments) and intervention tools that identify and suppress such PBs in parenting intervention programmes to prevent internalising problems. These should also include strategies to promote healthier PBs that encourage the development of various emotion regulation mechanisms in adolescents.

In addition to such programmes, there should also be included interventions especially aimed at developing different emotional regulation mechanisms in children and adolescents. These could be most effective on adolescents under overcontrolling parenting since its effect on rumination was shown to be weakened by high levels of effortful control [41]. The enhancement of such skills provides several adequate emotion regulation mechanisms to address different problems and prevents the exclusive reliance on inward thinking. Since the use of rumination is usually a response to negative affect characterised by a continuous focus on it [1, 2], these interventions should encourage the development of adequate emotional regulation mechanisms directed to problem-solving.

These suggestions should be taken more into consideration towards groups in higher risk of developing rumination when exposed to certain PBs (i.e. girls, low effortful control, high environmental sensitivity and high negative affect). Effortful control, environmental sensitivity and negative affect should be assessed when a client shows signs of persistent engagement in thoughts revolving negative emotion and is exposed to negative PBs.

In general, this review highlights the importance of parenting on the cognitive development of children and adolescents. The world is full of complex problems to be solved and if we maximise in children and adolescents the potential to engage in problem-solving and avoid increasing inaction as a response, typical of rumination, we might have a bigger chance at having those problems solved. Under this premise, investment in positive parenting and emotion regulation programmes is not a trivial but rather a necessary matter.
